# Antifouling and Antioxidant Properties of PVDF Membrane Modified with Polyethylene Glycol Methacrylate and Propyl Gallate

**DOI:** 10.3390/ma17081867

**Published:** 2024-04-18

**Authors:** Ting Wang, Jun Hu, Zhengchi Hou, Haijun Yang

**Affiliations:** 1 Shanghai Institute of Applied Physics, Chinese Academy of Sciences, Shanghai 201800, China; wangting@sinap.ac.cn (T.W.); hujun@sinap.ac.cn (J.H.); 2 University of Chinese Academy of Sciences, Beijing 100049, China; 3 Shanghai Synchrotron Radiation Facility (SSRF), Shanghai Advanced Research Institute, Chinese Academy of Sciences, Shanghai 201204, China; yanghj@sari.ac.cn

**Keywords:** polyvinylidene fluoride, antifouling, antioxidation, homogeneous radiation grafting, ultrafiltration membrane

## Abstract

In this study, molecules of propyl gallate (PG) and polyethylene glycol methacrylate (PEGMA) were covalently bonded via a transesterification reaction and subsequently grafted onto polyvinylidene fluoride substrates using a homogeneous radiation grafting technique. The enhancement of the membranes’ hydrophilicity with the increment of the grafting rate was corroborated by scanning electron microscopy imaging and measurements of the water contact angle. At a grafting degree of 10.1% and after a duration of 4 min, the water contact angle could decrease to as low as 40.1°. Cyclic flux testing demonstrated that the membranes modified in this manner consistently achieved a flux recovery rate exceeding 90% across varying degrees of grafting, indicating robust anti-fouling capabilities. Furthermore, these modified membranes exhibited significant antioxidant ability while maintaining antifouling performance over 30 days. The ability of the modified membranes to scavenge 2,2-diphenyl-1-picrylhydrazyl (DPPH) and 2,2′-Azino-bis(3-ethylbenzothiazoline-6-sulfonic acid) diammonium salt (ABTS^+^) free radicals remained nearly unchanged after being stored in pure water for 30 days, and the flux recovery rate remained above 95% after immersion in sodium hypochlorite solution for 30 days. Among the tested membranes, the PVDF-g-PEGMAG modified membrane with a grafting degree of 7.2% showed the best antioxidant effect.

## 1. Introduction

As economies grow and societies progress, the role of high-performance polymer filtration membranes gains prominence across sectors like water treatment and biomedicine [1,2,3,4,5,6,7,8]. Among these membranes, ultrafiltration membranes are indispensable components that face limitations due to fouling [9,10,11,12], which impairs performance and reduces lifespan due to oxidation during the cleaning phases [12,13,14]. Moreover, the demand for these membranes to possess antioxidant properties is critical, especially in the separation of bioactive substances to curb or eliminate oxidative stress triggered by reactive oxygen species in procedures like hemodialysis [15,16,17,18]. Therefore, the development of ultrafiltration membranes with enhanced antifouling and antioxidant features is of paramount importance.

In recent years, numerous studies have proposed various modification methods to alleviate membrane fouling issues, such as modification with inorganic ion exchangers, oxidation of graphene, metal–organic frameworks (MOFs), and others [19,20,21,22,23,24]. Polyvinylidene fluoride (PVDF) is a commonly used material for ultrafiltration membranes. There are many research works that highlight the significant improvement in fouling resistance when PVDF undergoes PEGylation, enhancing the performance of membranes fabricated from this material [25,26,27,28]. Wang et al. [25] employed the oxidative strength of ozone to activate PVDF, creating peroxides. This step was crucial for initiating the free radical graft polymerization process, which facilitated the grafting of polyethylene glycol methacrylate (PEGMA) onto the PVDF structure. Such a modification markedly improved the membrane’s capacity to repel protein adsorption. Through the utilization of Atom Transfer Radical Polymerization (ATRP), Hester [26] and Liu et al. [27] developed PVDF-g-PEGMA. Additionally, Liu et al. [27] successfully constructed a PVDF/PVDF-g-PEGMA filtration membrane with exemplary antifouling characteristics and devoid of any defects by incorporating the grafted polymer as a functional additive. Previous explorations by our research group [28] have demonstrated the efficacious grafting of methoxy polyethylene glycol acrylate (mPEGA) onto PVDF, employing it as a PEG monomer via a homogeneous radiation grafting technique. This grafted composite was directly utilized in membrane formation, showcasing a water flux recovery rate that consistently remained above 90% across three fouling cycles. Furthermore, there are studies that have also delved into improving the hydrophilicity and antifouling capabilities of PVDF membranes through the strategic incorporation of PEGylation during the surface grafting [29,30,31,32] and the membrane fabrication process [33], thus underscoring the importance of PEGylation in enhancing membrane functionality.

Enhancing a membrane’s hydrophilicity significantly improves its resistance to fouling [9,34]. However, maintaining these membranes through periodic cleaning is equally critical for extending their operational longevity and reducing expenses associated with their use [35,36,37,38,39,40]. In the realm of maintenance, chemical cleaning stands alongside physical methods as a vital approach, employing potent agents like hydrochloric acid (HCl), sodium hydroxide (NaOH), and sodium hypochlorite (NaClO), notable for their robust acidic, alkaline, and oxidizing effects, respectively [13,38,41,42,43,44,45,46,47,48,49,50]. NaClO is one of the most commonly used chemical agents in membrane cleaning processes [51]. The durability and functional lifespan of these membranes are heavily influenced by their capacity to withstand oxidation, making oxidation resistance a pivotal characteristic [52]. Investigative efforts by Gao et al. [43] have shed light on how different concentrations of sodium hypochlorite impact PVDF membranes, establishing guidelines for optimal cleaning practices to ensure filtration efficiency. Furthermore, Zhang et al. [34] have provided insights through comparative studies on the susceptibility of PVDF and polysulfone ultrafiltration membranes to damage and performance degradation when exposed to sodium hypochlorite solutions.

Derived from tannin hydrolysis, gallic acid (GA) is a polyphenolic compound with a spectrum of biological activities, including anti-inflammatory, antioxidant, and antibacterial properties [53,54,55,56,57,58,59]. In the realm of membrane technology, GA has been the focus of various studies. Employing a one-step dip-coating technique, Yang et al. [60] fabricated a hydrophilic PVDF membrane with GA and gamma-aminopropyltriethoxysilane, which exhibited remarkable hydrophilicity and antifouling characteristics. Through biomimetic coating, Cheng et al. [61] demonstrated the creation of a porous nanofiltration (NF) membrane via the Schiff base reaction, linking GA’s phenolic group with the amino group of polyethyleneimine. Additionally, Zhao et al. [62] synthesized a GA-modified polyethyleneimine layer on a hydrolyzed polyacrylonitrile base using a co-deposition method.

To enhance the antioxidative capabilities of filter membranes, numerous studies have integrated a variety of antioxidants directly into the membrane material [63]. Addressing the challenges associated with the migration of antioxidant molecules and their extraction by solvents during application, several researchers have explored chemically bonding antioxidants to the membrane surface [64,65]. However, reports on antioxidative modifications applied to PEGylated filter membranes remain absent from the literature.

In this study, a dual-functional PVDF membrane endowed with both antifouling and antioxidative properties was successfully developed. The initial phase involved a transesterification process between propyl gallate (PG) and PEGMA. Following this reaction, the incorporation of PVDF into the mixture led to the formation of PVDF-g-PEGMAG through a homogeneous radiation grafting method. The fabrication of ultrafiltration membranes was achieved using the technique of non-solvent-induced phase separation (NIPS). The degree of grafting (DG) was determined using the gravimetric method. Furthermore, the grafted product underwent molecular structural analysis through FTIR spectroscopy. Assessments of the membrane’s surface morphology, hydrophilicity, and filtration capacity were conducted utilizing SEM, water contact angle measurements, and water flux tests, respectively. The antifouling properties of the membrane were rigorously evaluated by filtering a solution of BSA and subsequently measuring the recovery rate of water flux following a cleansing process. The membrane’s antioxidant efficacy was determined through its ability to scavenge DPPH and APTS^+^ free radicals, with additional tests conducted to ascertain the longevity of these antioxidative characteristics by submerging the membrane in distilled water for an extended duration. Lastly, the resilience of the modified PVDF membrane against oxidative challenges was rigorously assessed by exposing it to a sodium hypochlorite solution over a specified timeframe, thereby evaluating its long-term oxidation resistance. This comprehensive study illustrates the potential of PVDF-g-PEGMAG membranes in applications requiring robust antifouling and antioxidative functionalities.

## 2. Materials and Methods

### 2.1. Materials

The PVDF powder (Solef 6020, Mn = 670,000) was purchased from Solvay Co., Ltd. (Qingdao, China). Polyethylene glycol methyl acrylate (PEGMA), 2,2-diphenyl-1-picrylhydrazyl (DPPH), and 2,2′-Azino-bis(3-ethylbenzothiazoline-6-sulfonic acid) diammonium salt (ABTS^+^) were obtained from Sigma-Aldrich Trading Co., Ltd. (Shanghai, China). Propyl gallate (PG), p-toluenesulfonic acid (TsOH), N,N-dimethylformamide (DMF), polyvinylpyrrolidone (PVP-K30), sodium chloride (NaCl), disodium hydrogen phosphate (Na_2_HPO_4_), potassium chloride (KCl), potassium dihydrogen phosphate (KH_2_PO_4_), hydrochloric acid (HCl), sodium hypochlorite solution (NaClO), bovine serum albumin (BSA), anhydrous ethanol, and potassium persulfate (K_2_S_2_O_8_) were purchased from Sinopharm Chemical Reagent Co., Ltd. (Shanghai, China). The PVDF powder was washed three times with ultrapure water before use and dried at 80 °C for 24 h in a vacuum oven. All chemical reagents were used without further purification. Additionally, water purified through a Milli-Q system from Millipore was employed.

### 2.2. Synthesis of Graft Copolymer

**Figure 1:**
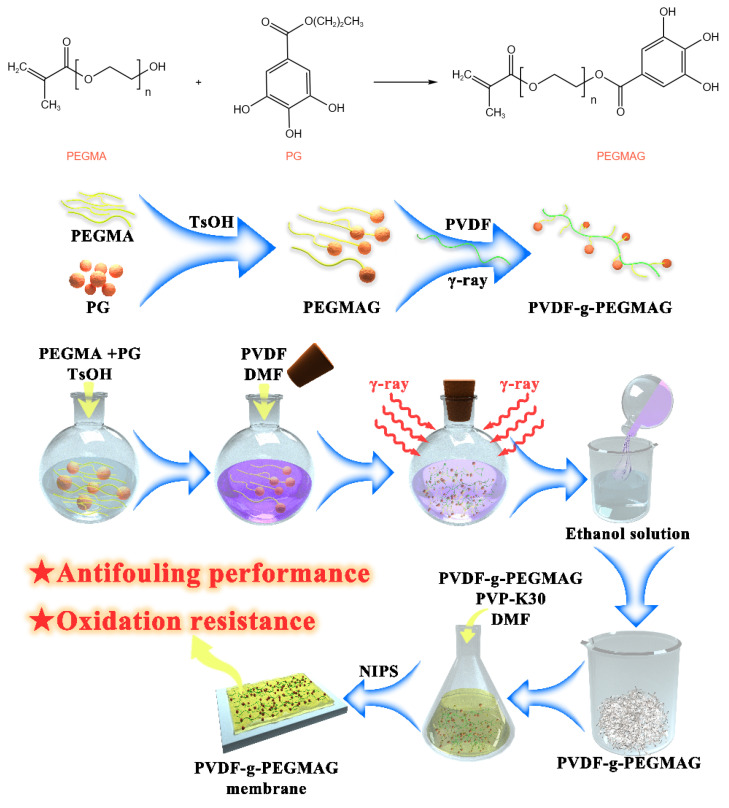
Schematic diagram of the synthesis/fabrication process.

A mixture of PEGMA and TsOH was placed into a reaction flask, subsequently heated to 120 °C in an oil bath with ongoing stirring. Following this, PG was incrementally introduced to the mixture while stirring persisted. This reaction was conducted over a span of four hours, with vacuum application for one minute at each hour to eliminate volatiles. Once the reaction phase concluded and the mixture had cooled, PVDF and DMF were added, followed by stirring at 70 °C for a full day to achieve a homogenous solution. After cooling to ambient temperature, nitrogen gas was purged into the flask for thirty minutes before sealing. The samples were then subjected to γ-radiation from a ^60^Co source, with a dose of 20 kGy for 18 h at room temperature. After irradiation, the solution was slowly transferred into an ethanol solution for precipitation. Through repeated rinsing and washing in ethanol, unreacted substances and homopolymers were removed. The final step involved drying the precipitate in a vacuum oven at 80 °C until reaching a constant weight to yield PVDF-g-PEGMAG. The reagent quantities used are specified in Table 1. The schematic diagram of the study is shown in Figure 1.

The degree of grafting (DG) of PVDF-g-PEGMAG is defined in Equation (1):

(1) $$DG=\frac{W_{1}-W_{0}}{W_{0}}\times 100\%$$

where *W*_1_ denotes the mass of PVDF-g-PEGMAG and *W*_0_ is the mass of pristine PVDF.

### 2.3. Fourier Transform Infrared Spectroscopy Characterization

ATR-IR spectroscopy analyses were conducted for pristine polyvinylidene fluoride (PVDF), PVDF-g-PEGMA, and PVDF-g-PEGMAG samples utilizing a Nicolet Avatar 370 infrared spectrometer (Thermo Nicolet Instrument Corporation, Madison, WI, USA). The spectral data collection covered a wavelength range from 400 cm^−1^ to 4000 cm^−1^ with a resolution set at 4 cm^−1^. Each spectrum acquisition involved 32 repetitive scans to ensure accuracy. Given the challenges associated with pulverizing the grafted polymer, a membrane approach was adopted for preparing the samples, consistent with methodologies described in preceding research [28].

### 2.4. Preparation of Membrane

The membranes were fabricated using the non-solvent-induced phase separation (NIPS) method. The composition of the casting solution encompassed a polymer concentration of 15 wt.%, a porogen PVP-K30 at 4 wt.%, and the solvent DMF constituting 81 wt.%. Utilization of a casting knife facilitated the even distribution of the casting solution across a glass plate, targeting a uniform film thickness of 200 μm. The solution underwent brief evaporation in the ambient atmosphere for 15 s, followed by immersion in deionized water. To assure the thorough elimination of any residual solvents, the membrane was meticulously preserved in water for a duration of two days, during which the water was refreshed multiple times before any testing commenced.

### 2.5. Dynamic Water Contact Angle Measurement

Contact angle measurements of the membrane were carried out using the Attension Theta system (KSV Instruments Ltd., Helsinki, Finland). For this process, droplets, each measuring 5 μL, were carefully dispensed onto the membrane’s surface from a needle’s tip. Over a 4 min duration, a digital camera captured the interaction between the droplet and the membrane surface in video format. Subsequently, specialized software in the system analyzed these video recordings to determine the contact angle values.

### 2.6. Analysis of Scanning Electron Microscope

Morphological analysis of the membrane was conducted using a LEO1530vp SEM (Zeiss, Jena, Germany). To prepare the membrane for examination, it was first subjected to rapid freezing in liquid nitrogen, followed by a brittle fracture to reveal the cross-section. The images obtained were then analyzed with ImageJ software (National Institutes of Health, Bethesda, MD, USA, v1.8.0), allowing for the quantification of pores, as well as the measurement of pore size and distribution across a given unit area.

### 2.7. Filtering Experiment

**Figure 2:**
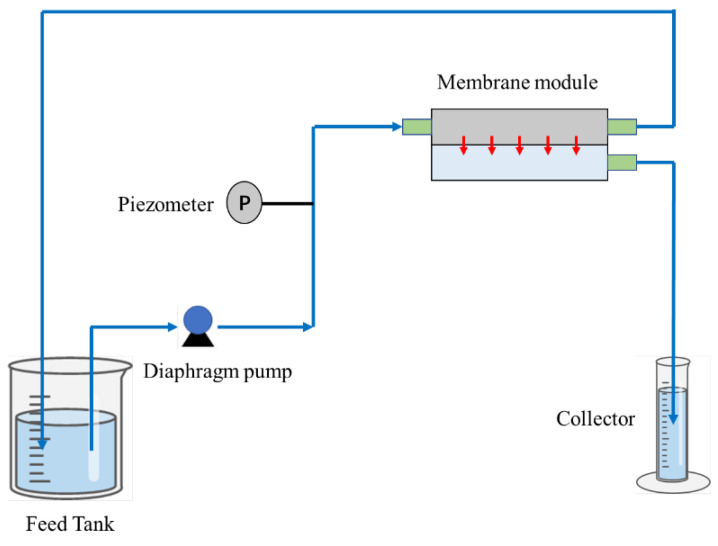
The schematic diagram of the cross-flow device used in the filtration experiment [28].

The membrane’s performance was assessed using the identical cross-flow apparatus utilized in prior experiments, as depicted in Figure 2 [28]. Each membrane was fashioned into a circular specimen boasting a 15.9 cm^2^ surface area and underwent a pre-compression process at 0.1 MPa for a duration of 20 min. For each type of membrane, a set of three independent trials was conducted. The flux measurement involved the quantification of the solution volume passing through the membrane over a specified period, subsequently normalized to the membrane’s surface area.

The water flux (*F*_0_) was calculated according to Equation (2):

(2) $$F_{0}=\frac{Q}{A∆T}$$

where *Q* refers to the volume of the filtered water; *A*, the area of the membrane; and Δ*T*, the time of the filtration.

In this study, a BSA buffer solution at a concentration of 1 g/L was selected as the model for pollutant testing, employing the same buffer preparation protocol established in preceding experiments. Samples were collected both before and after the filtration process to facilitate subsequent analysis. The quantification of BSA concentration within the solution was accurately performed using a UV-1100 visible ultraviolet spectrophotometer (Beijing Ruili Instrument Co., Ltd., Beijing, China), in accordance with methodologies cited in the reference literature [66].

### 2.8. Evaluation of Antifouling Performance of Membrane

The evaluation of the membrane’s antifouling capability was conducted by measuring the pure water flux recovery rate following the filtration of a BSA solution. In the cross-flow filtration setup, the feed solution was alternated between pure water and the BSA buffer solution every 60 min. After completing three cycles of this process, the recovery rate of water flux through the membrane was determined.

The flux recovery ratio (*FRR*) is calculated according to Equation (3):

(3) $$FRR=\frac{F_{1}}{F_{0}}\times 100\%$$

where *F*_0_ and *F*_1_ denote the water flux prior to and following fouling, respectively.

### 2.9. Evaluation of the Oxidation Resistance of the Membrane

The antioxidative properties of the membrane were evaluated from two perspectives. Firstly, the membrane’s ability to scavenge DPPH and ABTS^+^ radicals was assessed. Secondly, the membrane’s resistance to oxidation in NaClO solution was evaluated.

#### 2.9.1. Free Radical Scavenging Effect

A solution of DPPH (0.1 mM) was formulated using anhydrous ethanol. Subsequently, membrane samples, each measuring 1 × 1 cm^2^, were submerged in 3 mL of this DPPH solution and left to incubate for 1 h in a dark environment at room temperature. As a control, a DPPH solution without any membrane sample underwent identical treatment. The absorbance of these solutions was then recorded at a wavelength of 517 nm.

To prepare the ABTS^+^ radical cation solution (0.1 mM), a mixture of ABTS^+^ solution (2 mL, 7 mM) and potassium persulfate (K_2_S_2_O_8_) solution (2 mL, 2.45 mM), diluted with pure water, was allowed to stand for 12 h in the dark at 4 °C. This solution was subsequently adjusted with anhydrous ethanol to achieve an absorbance of 0.700 ± 0.025. Each membrane sample, again measuring 1 × 1 cm^2^, was immersed in 4 mL of the ABTS^+^ solution and left for 20 min in a dark environment at room temperature. The absorbance of this solution was measured at 734 nm.

Equation (4) for calculating the scavenging rate of the membranes against these two free radicals is presented as follows:

(4) $$Scavenging=\frac{A_{0}-A_{1}}{A_{0}}\times 100\%$$

where *A*_0_ represents the absorbance of the control sample devoid of the membrane, while *A*_1_ signifies the absorbance recorded for the solution containing various membrane samples.

The enduring stability of antioxidant capabilities holds considerable relevance for the practical application of antioxidant membranes. To assess this, membrane samples measuring 1 × 1 cm^2^ were submerged in distilled water for a duration of 30 days. The efficiency of these membranes in scavenging free radicals was determined at intervals—on the 1st, 7th, 14th, and 30th days, to gauge the long-term retention of their antioxidant attributes.

#### 2.9.2. Oxidation Resistance of Membrane to NaClO Solution

Initially, a variety of membranes were submerged in a NaClO solution with a concentration of 0.2 wt% for a duration of 30 days, with the NaClO solution being refreshed daily to maintain consistent concentration levels. The performance of each membrane, in terms of water flux and BSA rejection rates, was documented on the 1st, 7th, 14th, and 30th days. Furthermore, a modified membrane, possessing a DG of 8.6%, was also immersed in the NaClO solution for 30 days. Following this period, it was thoroughly rinsed with pure water, and a circulation flux test was conducted. The resistance of the membrane to oxidation by NaClO solution was assessed based on these two investigative approaches.

## 3. Results and Discussion

### 3.1. FTIR Spectra of Graft Copolymer

**Figure 3:**
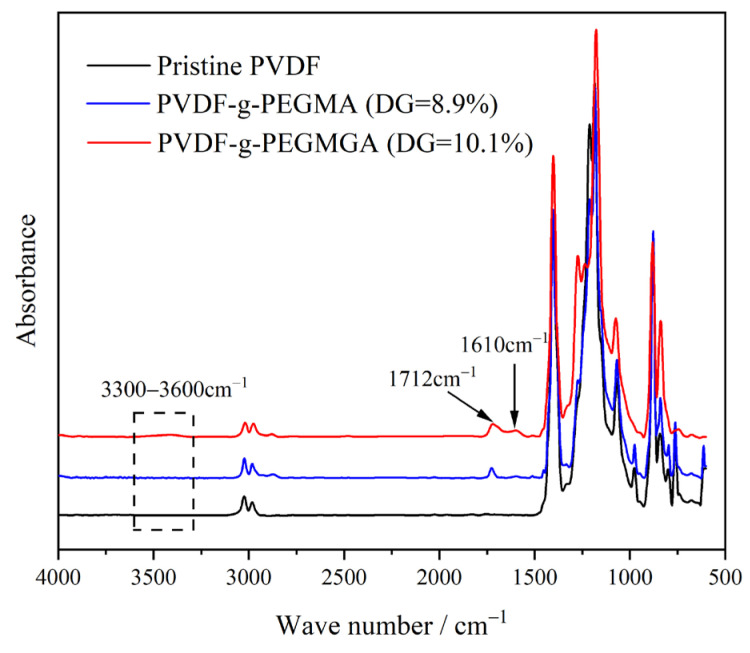
FTIR spectra of pristine PVDF and different grafted polymers.

Figure 3 illustrates the infrared spectra of the pristine PVDF alongside various grafted polymers, following vector normalization. Among these, PVDF-g-PEGMA, with a DG of 8.9%, was synthesized by directly grafting PEGMA onto PVDF using the previously described homogeneous radiation grafting technique [28]. Notably, both grafted polymers displayed a new characteristic absorption peak at 1712 cm^−1^, indicative of the carbonyl (C=O) group present in PEGMA [67]. When comparing PVDF-g-PEGMA to PVDF-g-PEGMAG, the latter exhibited subtle signals corresponding to phenolic hydroxyl groups and benzene rings, characteristic of PG molecules, in the range of 3300–3600 cm^−1^ and at around 1610 cm^−1^, respectively [68]. These spectral features confirm the successful grafting of PEGMA and PG onto the PVDF chain, resulting in the synthesis of PVDF-g-PEGMAG.

### 3.2. Dynamic Water Contact Angle Test

**Figure 4:**
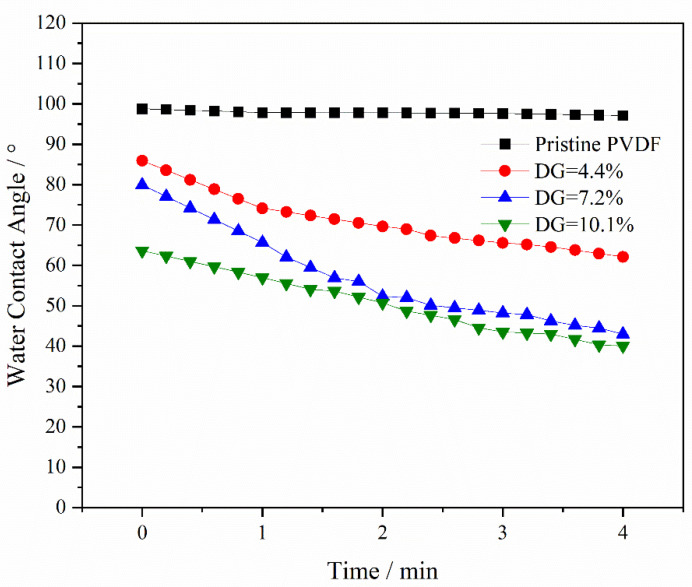
Dynamic water contact angles of pristine PVDF and PVDF-g-PEGMAG membranes with different DG.

Figure 4 displays the dynamic water contact angles of both the pristine PVDF and the PVDF-g-PEGMAG membranes with varying degrees of grafting. The water contact angle for the unmodified PVDF remained consistently above 95°, indicating pronounced hydrophobicity. For the PVDF-g-PEGMAG membranes, an increase in the degree of grafting correlated with an increase in the water contact angle, signifying enhanced hydrophilicity and, consequently, improved surface wetting. Notably, at a grafting degree of 10.1% and after a duration of 4 min, the water contact angle could decrease to as low as 40.1°. This demonstrates a substantial enhancement in hydrophilicity for the grafted polymers in comparison to the pristine PVDF. It was observed that the addition of PG segments to the PEGylated PVDF materials did not alter their hydrophilic properties.

### 3.3. Morphology Characterization of Membrane

**Figure 5:**
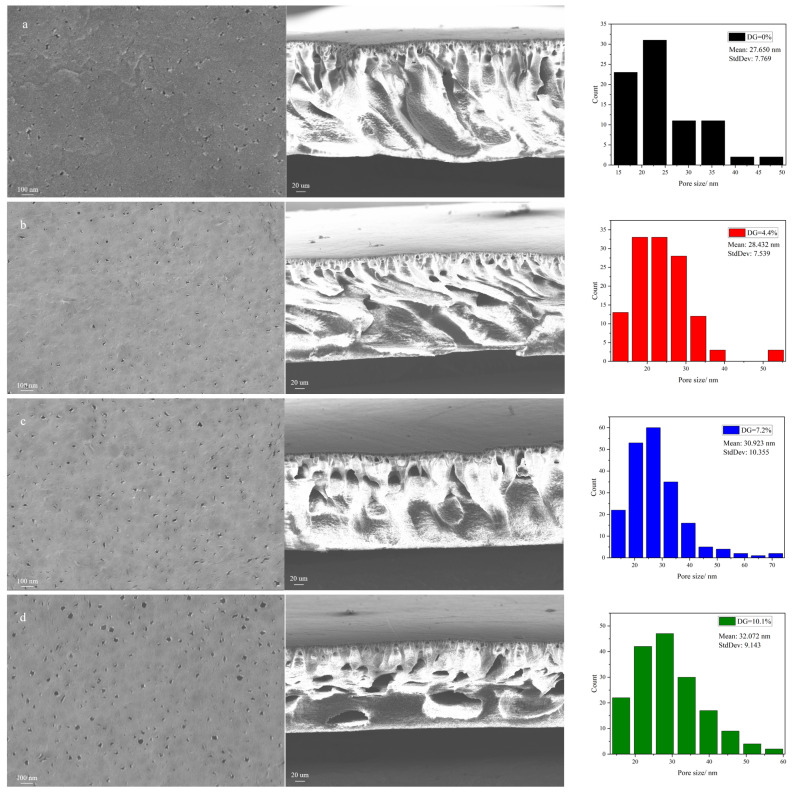
SEM images of membrane surface and cross-section with different grafting ratios: (**a**) DG = 0, (**b**) DG = 4.4%, (**c**) DG = 7.2%, (**d**) DG = 10.1%. (The rightmost is the square distribution of the membrane pore size calculated by ImageJ based on the SEM membrane surface image).

Figure 5 presents the surface and cross-sectional images of membranes with varying grafting ratios, alongside a pore size distribution histogram derived from the surface images using ImageJ software (v1.8.0). The images reveal that each membrane exhibits an asymmetric structure characterized by finger-like pores. The membrane surface pore sizes are 27.650 nm, 28.432 nm, 30.923 nm, and 32.072 nm in Figure 5a–d, respectively. With an increase in grafting ratio, there is a noticeable enlargement in pore size on the membrane surface, and the cross-sectional views begin to show larger pores more prominently [69,70]. In general, an NIPS process generates a dense skin and porous sub-layer. The dense surface is formed due to the solidification of the polymer top layer induced by a fast solvent outflow, and the porous sub-layer is induced from the liquid–liquid demixing where the solution phase separates into a polymer-lean phase and a polymer-rich phase [71]. The pore formation in the NIPS process may be attributed to the rapid diffusion of solvent into the non-solvent (water), while the non-solvent does not permeate as swiftly through the interface between the casting solution and water, generating significant tension within the final membrane matrix, thus forming the distinctive finger-like pores. As the grafting ratio rises, the diffusion rate of the non-solvent (water) accelerates, making the macroscopic pore structure in the membrane matrix more pronounced in the cross-sectional area [72]. Moreover, at the same polymer weight percentage, a higher grafting ratio implies a greater content of grafting segments and, consequently, a lower concentration of pure PVDF in the polymer. This results in a quicker solvent diffusion rate and, subsequently, larger pores in the modified membrane [73].

### 3.4. Evaluation of Filtration Performance and Antifouling Performance of Membrane

**Figure 6:**
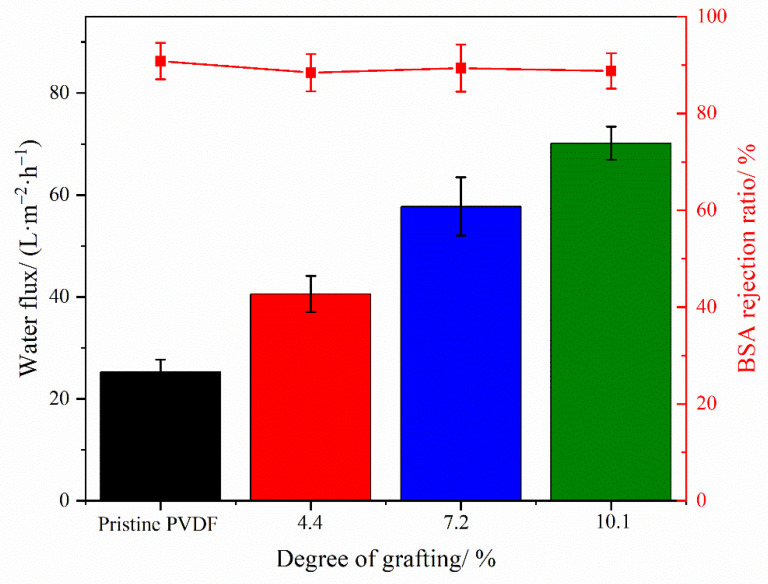
Water flux and BSA rejection of polymer membranes with different DG.

Figure 6 illustrates the comparison of water flux and BSA rejection between the pristine PVDF membranes and those modified with PVDF-g-PEGMAG at various DG. The graph demonstrates that, as the DG increases, so does the water flux through the membrane. This observation aligns with findings from previous contact angle tests, which indicated an enhancement in membrane hydrophilicity concurrent with rising DG levels. Enhanced hydrophilicity leads to diminished filtration resistance, thereby facilitating increased flux. Additionally, this elevation in water flux corresponds with the morphological attributes identified in the membrane, such as the expansion in pore size on the membrane surface and the emergence of larger pores within the membrane cross-section. The BSA rejection rates for all the membranes hovered around 90%, indicating the effectiveness of the modified membranes, crafted using grafted polymers, in maintaining robust filtration performance.

**Figure 7:**
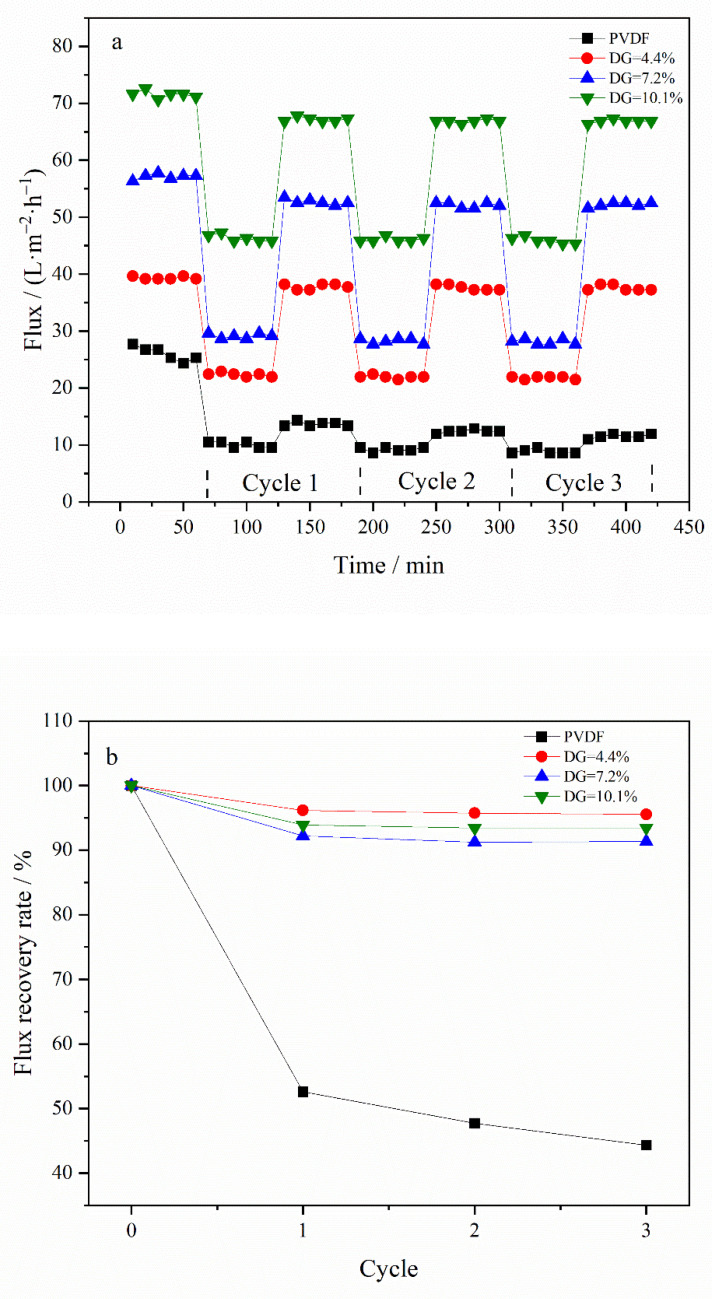
Antifouling cycle test of membrane: (**a**) water and BSA flux of membrane with different DG under multiple cycles, (**b**) FRR of membrane with different DG under multiple cycles.

In this study, an in situ measurement technique was employed, integrating online cleaning of the membrane via a cross-flow filtration method. This process entailed alter-nating the fluid composition, specifically switching between water and a BSA solution. Figure 7 illustrates the results from antifouling cycle tests on membranes with varying DG. Specifically, Figure 7a depicts the fouling and cleaning flux of the membrane across multiple cycles, whereas Figure 7b presents the FRR derived from the data in Figure 7a. During the cleaning phase, consistent trends in flux variation were observed among different modified membranes, with a notable increase in flux upon switching the feed to pure water. After three cycles (420 min) of testing, the flux of pure water post-cleaning remained relatively unchanged, with only a minor reduction observed during the initial cycle. Conversely, the pristine PVDF membrane exhibited a significant flux decline upon the initial switch to pure water. Furthermore, the FRR values calculated from the flux data indicated a significant decrease in the FRR of the pristine PVDF membrane during the first cycle, with a continued decline over the subsequent two cycles. In contrast, the FRRs for the modified membranes consistently exceeded 90% and remained stable through later cycles, suggesting that higher FRR values correlate with enhanced membrane stability and superior antifouling performance. The antifouling cycle tests comprehensively demonstrate the superior antifouling capabilities of the modified membranes.

### 3.5. Oxidation Resistance of the Membrane

#### 3.5.1. Free Radical Scavenging Effect of Membrane

**Figure 8:**
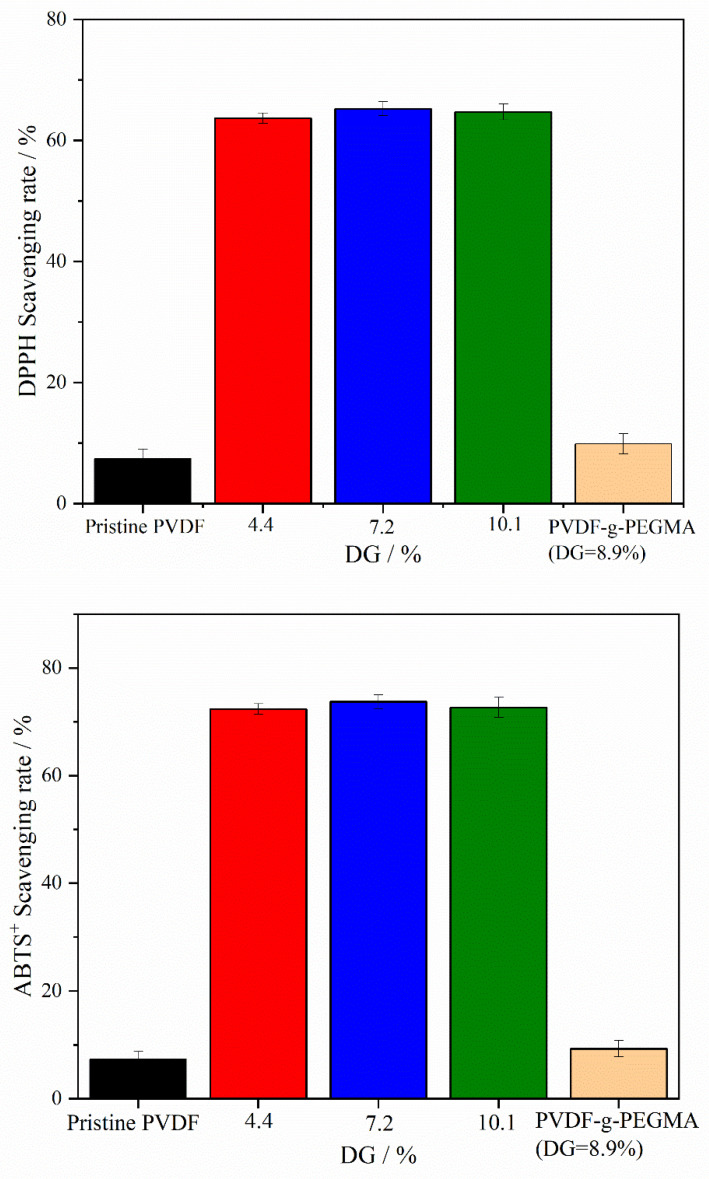
Scavenging rates of DPPH and ABTS^+^ free radicals by different types of membranes.

Figure 8 demonstrates the efficiency of different types of membranes in scavenging DPPH and ABTS^+^ free radicals. The membranes modified with PG segments, PVDF-g-PEGMAG, showcased a significantly higher free radical scavenging capacity compared to both the pristine PVDF and the PVDF-g-PEGMA. This superior scavenging ability highlights the impactful role of the PG segments in enhancing the membrane’s antioxidative properties. Furthermore, variations in the grafting rate appeared to have a minimal impact on the membranes’ ability to neutralize free radicals. This observation could be attributed to the fact that the grafting rate mentioned in this study reflects the combined grafting of both PG and PEGMA segments onto the membrane. During the synthesis of the grafted polymer with a DG of 10.1%, a larger quantity of PEGMA segments was introduced onto the PVDF chain compared to PEGMAG, due to the higher proportion of PEGMA, thereby leading to a slight decrease in the membrane’s free radical scavenging rate compared to the membrane with DG = 7.2%.

**Figure 9:**
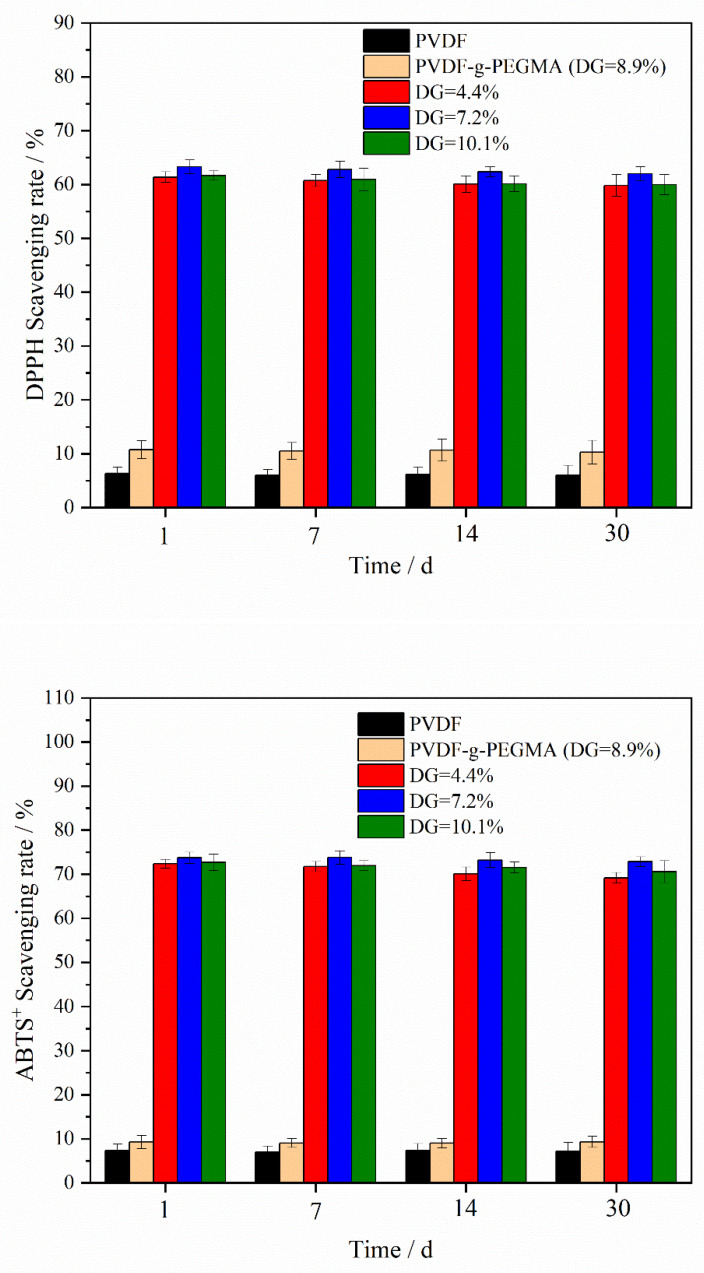
Scavenging stability of DPPH and ABTS^+^ free radicals by different types of membranes. (Membrane samples were submerged in distilled water for a duration of 30 days).

Figure 9 illustrates the durability of DPPH and ABTS^+^ free radical scavenging by various membrane types. The stability of the membrane’s antioxidative performance is a parameter that needs to be considered during the normal usage of the membrane. In this investigation, the antioxidant stability of the membranes was assessed based on their ability to neutralize free radicals after being submerged in pure water for an extended duration. The data indicate that the scavenging efficiency for both types of free radicals diminished only marginally after a 30-day immersion period in pure water. This experiment underscores that the modified membranes possess not just a measure of resistance to oxidation, but also maintain considerable stability in their antioxidative properties over time.

#### 3.5.2. Oxidation Resistance of Membrane to NaClO Solution

**Figure 10:**
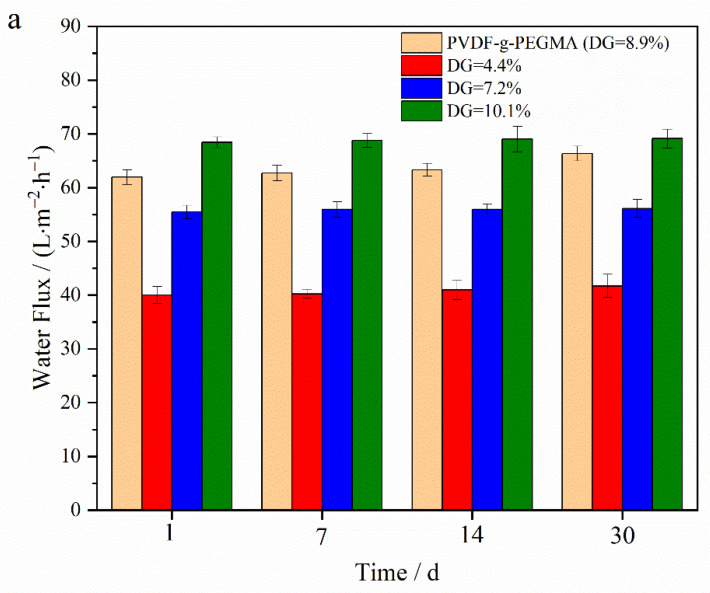

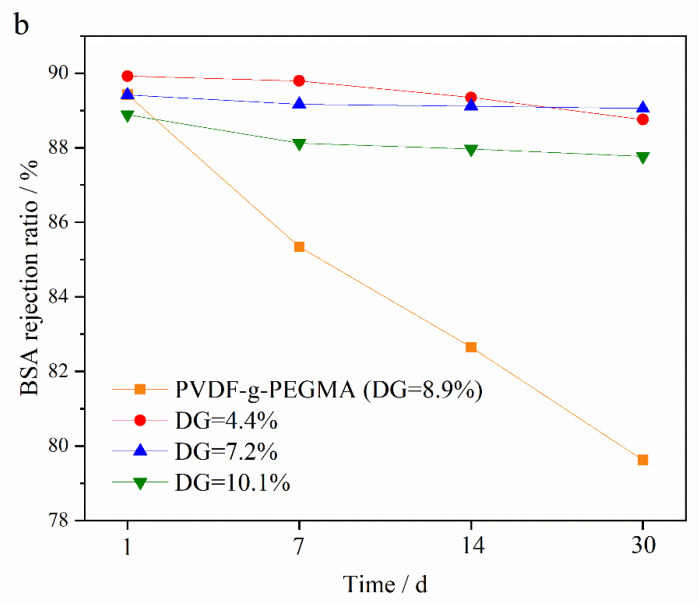
Water flux (**a**) and BSA rejection (**b**) of different types of membranes immersed in NaClO (2 wt.%) solution for a period of time.

**Figure 11:**
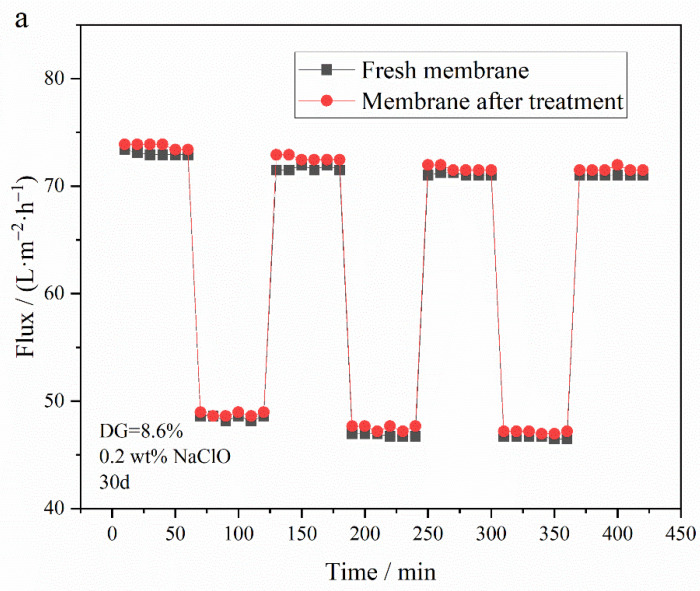

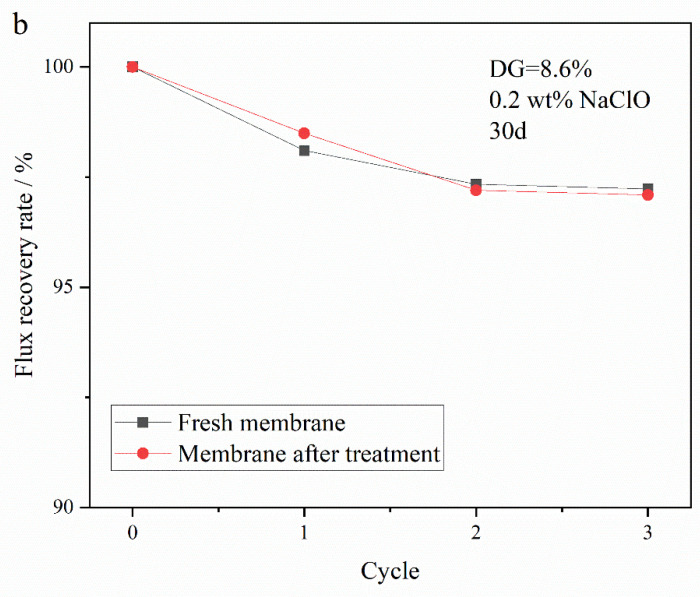
Antifouling cycle test of PVDF-g-PEGMAG (DG = 8.6%) membrane before and after immersion in NaClO (0.2 wt.%) solution for 30 days: (**a**) water and BSA flux under multiple cycles before and after oxidation, (**b**) FRR under multiple cycles before and after oxidation.

To investigate the influence of an oxidizing agent on membranes post-grafting of PG segments, membranes, including PVDF-g-PEGMA (with a DG of 8.9%) and PVDF-g-PEGMAG with various DG, were immersed in a sodium hypochlorite (NaClO) solution (2 wt.%) for a set duration. The impact on membrane filtration performance was assessed through variations in water flux and bovine serum albumin (BSA) rejection rate over time. Previous reports on the effect of hypochlorite treatment on UF membranes indicated a flux increase in NaClO-treated membranes [12,14]. Specifically, the PVDF-g-PEGMAG membrane with a DG of 8.6% was analyzed to compare its circulation flux before and after a 30-day immersion in NaClO solution. The antifouling efficacy of the membrane was appraised by comparing the FRR before and after the 30-day exposure to oxidation. Figure 10 reveals that, after 30 days of exposure to the oxidizing environment, the water flux through the PVDF-g-PEGMA membrane increased, while its BSA rejection rate dropped below 80%, indicating a significant detriment to its filtration performance due to oxidation. Conversely, the water flux and BSA rejection rates for PVDF-g-PEGMAG membranes with various DG remained largely stable after the 30-day oxidation period. This suggests that the PG segment grafting effectively mitigated the oxidation impact on PEGylated PVDF membranes. Figure 11 displays the antifouling cycle test results for the PVDF-g-PEGMAG membrane with a DG of 8.6%, both before and after undergoing oxidation treatment for 30 days. It is observed that the flux of the membrane post-oxidation closely mirrors the FRR prior to oxidation, maintaining stability across three cycles. This outcome indicates that the antifouling properties of the modified membrane were well-preserved even after 30 days of exposure to oxidizing conditions.

## 4. Conclusions

In this study, the PG segment was initially bonded to the PEGMA molecule through a transesterification process. Subsequently, this complex was grafted onto PVDF using a homogeneous radiation grafting technique to create PVDF-g-PEGMAG, which incorporates both antioxidant (PG segment) and antifouling (PEGMA segment) functionalities.

The membrane of the grafted polymer was fabricated by employing the NIPS method. Analysis of the membrane’s surface and cross-section via SEM alongside water contact angle measurements revealed that the hydrophilicity of the membrane enhanced as the DG increased. The pore size of the membrane increased with the increase in grafting rate. Specifically, for the modified membrane with a grafting rate of 10.1%, the pore size expands from 27.650 nm in the original PVDF membrane to 32.072 nm. Additionally, after a duration of 4 min, the water contact angle of the membrane could decrease to as low as 40.1°. Cyclic flux testing of the membrane indicated that introducing the PG segment to the modified membrane did not detract from the excellent antifouling capabilities inherent to PEGylated PVDF membranes. In tests of circulation flux across membranes with varying DG, the FRR consistently exceeded 90%. Moreover, the antioxidant performance tests demonstrated that the modified membrane retained its capacity to scavenge DPPH and ABTS^+^ free radicals, with its efficacy largely unaffected by prolonged immersion in water. After soaking in pure water for 30 days, the modified membrane exhibited only a slight decrease in scavenging rates for both DPPH and ABTS^+^ free radicals, maintaining them at above 60% and 70%, respectively. Additionally, the PG grafting significantly bolstered the membrane’s resistance to oxidation, enabling it to maintain antifouling performance; even after a 30-day immersion in a NaClO solution, the flux recovery rate remained above 95%, thus illustrating the membrane’s durability against oxidation during chemical cleaning processes.

This study successfully developed a membrane that exhibits both antifouling and antioxidative properties, highlighting its long-term effectiveness and stability. Although further research is needed to assess the membrane’s tolerance to other chemical cleaning methods, such as acid–base treatments, to differentiate the proportion of functional components in the grafted product, and to elucidate the mechanism of antioxidation. However, the simplicity and potential scalability of the membrane fabrication process make it a viable option from an industrial perspective. The membrane exhibits promising potential for applications in water treatment and the membrane-based separation of bioactive substances.

## Figures and Tables

**Table 1:** The formula for synthesizing PVDF-g-PEGMAG.

No.	PEGMA/g	TsOH/g	PG/g	PVDF/g	DMF/g	Total/g
1	3	0.08	4.24	5	37.68	50
2	5	0.08	7.07	5	32.85	50
3	9	0.08	12.72	5	24	50

## Data Availability

Data are contained within the article.
